# MoCA Cognition Digital Platform Supports Brain Health in the Community

**DOI:** 10.1002/alz70858_103705

**Published:** 2025-12-24

**Authors:** Ziad Nasreddine, Thomas TANNOU, Paolo Vitali, Felix Pageau, Marie Christine Le Bourdais, Guy Lacombe, Murray Gilles, Laura Klaming, Willem Huijbers

**Affiliations:** ^1^ MoCA Clinic and Institute, Greenfield Park, QC, Canada; ^2^ CRIUGM, CIUSS Centre‐Sud de l'Ile de Montréal, Montreal, QC, Canada; ^3^ The McGill University Research Centre for Studies in Aging, Montreal, QC, Canada; ^4^ Université Laval, Québec, QC, Canada; ^5^ Alzheimer Society of Montreal, Montreal, QC, Canada; ^6^ Sherbrooke University, Sherbbrooke, QC, Canada; ^7^ CIUSSS de l'Estrie‐CHUS, Sherbrooke, QC, Canada; ^8^ MoCA Cognition, Montreal, QC, Canada

## Abstract

MoCA Cognition has developed an innovative digital platform designed to empower individuals to assess and monitor their cognitive performance through the XpressO Test by MoCA. This user‐friendly platform offers a comprehensive tool for early detection and intervention in cognitive decline, allowing users to track changes in their cognitive abilities over time. The XpressO Test provides a fast, reliable evaluation that measures key cognitive functions, enabling individuals to better understand their brain health and take proactive steps in maintaining it.

One of the platform's standout features is its Brain Health Score, which is based on the 2024 Lancet‐modifiable risk factors for cognitive decline. This score offers a personalized measure of brain health, considering various factors such as diet, exercise, sleep, and cognitive and social engagement. By quantifying these elements, the Brain Health Score provides valuable insights into areas that may require attention, offering actionable guidance to improve cognitive well‐being.

In addition to the cognitive assessment, the platform provides recommendations for lifestyle changes that can help reduce the risk of cognitive decline. These lifestyle interventions may include suggestions for improving sleep quality, increasing physical activity, adopting healthier dietary habits, and engaging in activities that stimulate the mind. By addressing these modifiable risk factors, users enhance their brain health and potentially delay or prevent cognitive decline.

The platform includes a medical questionnaire designed to identify underlying medical conditions or factors that may be influencing cognitive performance. This includes the impact of poor sleep, high stress, depression, and the use of certain psychotropic medications. By identifying these potential factors, the platform enables users to seek targeted interventions from healthcare providers, optimizing their chances for improved cognitive health.

Overall, MoCA Cognition's digital platform offers a valuable, accessible resource for individuals seeking to monitor and enhance their brain health, providing early detection, and proactive steps to maintain cognitive well‐being. The advantages of this platform align with the strategy of our collaborative group. It not only enhances the quality and accessibility of care but also empowers communities to take an active role in supporting individuals with neurocognitive disorders, ultimately improving their quality of life and care outcomes.

